# Enhancing Prostate Cancer Diagnosis: A Comparative Analysis of Combined Fusion and Systematic Biopsy Methods—A Single-Center Study

**DOI:** 10.3390/jcm14082822

**Published:** 2025-04-19

**Authors:** Emil Kania, Maciej Janica, Grzegorz Hrehoruk, Przemysław Kurowski, Adam Ostasiewicz, Paweł Samocik, Robert Kozłowski, Jacek Robert Janica

**Affiliations:** 1 Department of Urology, Śniadeckiego Voivodeship Hospital in Bialystok, 26 Sklodowskiej-Curie St., 15-278 Bialystok, Poland; 2 Department of Radiology, Śniadeckiego Voivodeship Hospital in Bialystok, 26 Sklodowskiej-Curie St., 15-278 Bialystok, Poland; 3 Department of Paediatric Radiology, Medical University of Bialystok, 17 Waszyngtona St., 15-274 Bialystok, Poland

**Keywords:** prostate cancer, fusion biopsy, combined prostate biopsy, systematic biopsy

## Abstract

**Background/Objectives:** Prostate cancer is the second most prevalent malignancy in men globally, typically suspected following abnormalities found during digital rectal examination (DRE) or elevated PSA levels. This study aimed to evaluate and compare the effectiveness of two biopsy techniques—TRUS-Bx and ComBx—in detecting prostate cancer, particularly focusing on the identification of clinically significant prostate cancer (csPCa) with an International Society of Urological Pathology (ISUP) grade ≥ 2. **Methods:** This retrospective cohort study involved 500 men (aged 46 to 79, with an average age of 65) who had prostate biopsies at our institution between 2017 and 2022. The patients were divided into two groups: 250 men received a transrectal US-guided biopsy (TRUS-Bx) with a standard 12-core sampling, while the other 250 underwent a transperineal combined fusion biopsy (ComBx) with MRI guidance. The ComBx group targeted areas classified as PI-RADS ≥ 3 and also included additional systematic samples. Tumor detection rates for both techniques were assessed, with a particular focus on overall PCa detection and the identification of csPCa. **Results:** In the TRUS-Bx group, the mean PSA level was 8.2 ng/mL (1.8–45.2 ng/mL), and in the ComBx group, the mean PSA level was 7.5 ng/mL (0.8–32.4 ng/mL). ComBx demonstrated superior detection rates for PCa compared to TRUS-Bx, with statistically significant differences observed in the overall detection of PCa with ISUP grade ≥ 1 (61% for ComBx vs. 45% for TRUS-Bx; *p* < 0.001) and csPCa (40% for ComBx vs. 30% for TRUS-Bx; *p* = 0.019). In the ComBx group for csPCa, the detection rates in targeted biopsies of MRI-identified lesions assessed as PIRADS 3, 4, and 5 were 17%, 51%, and 78%, respectively. **Conclusions:** ComBx offers significantly improved efficacy in detecting prostate cancer, particularly in identifying clinically significant cases, compared to systematic TRUS-Bx. These findings support ComBx as a valuable tool in enhancing diagnostic accuracy for csPCa.

## Simple Summary

Prostate cancer (PCa) is one of the most common cancers in men, often suspected when a physical exam reveals abnormalities or when blood tests show high levels of prostate-specific antigen (PSA). To confirm prostate cancer, a biopsy must be performed, where small samples of prostate tissue are taken for analysis. In a study involving 500 men aged 46 to 79, two prostate biopsy techniques were compared: the conventional transrectal ultrasound-guided biopsy (TRUS-Bx) and a novel approach known as combined fusion biopsy (ComBx). The latter integrates targeted biopsy samples (TBxs) and systematic biopsy samples (SBxs), utilizing both ultrasound and multiparametric magnetic resonance imaging (mpMRI) guidance. Results showed that ComBx was more effective at detecting prostate cancer, especially clinically significant types, compared to TRUS-Bx. Specifically, ComBx found cancer in 61% of cases compared to 45% with TRUS-Bx, and detected clinically significant cancer in 40% of cases versus 30%, respectively.

## 1. Introduction

Prostate cancer (PCa) is the second most commonly diagnosed cancer in men worldwide, with the highest incidence rates in North and South America, Europe, Australia, and the Caribbean. PCa remains the leading cause of cancer-related deaths in men across many countries, with an estimated 375,000 deaths globally in 2020 [1]. In Poland, PCa is among the most commonly diagnosed malignancies in men. In 2021, 17,832 cases and 5458 deaths were recorded, putting PCa at the top of the list of cancer diagnoses among men, with the number of deaths expected to rise to 6550 by 2030 [2,3]. Survival rates for PCa vary widely depending on the disease stage at diagnosis, with a 5-year survival rate approaching 100% for localized cases, compared to only 32.3% (31.6–33%) for metastatic disease [4]. These statistics highlight the need for effective diagnostic methods to accurately detect PCa and determine its stage at diagnosis.

PCa suspicion typically arises from abnormalities detected during digital rectal examination (DRE) or elevated prostate-specific antigen (PSA) levels. However, DRE is a subjective and operator-dependent test, with its accuracy influenced by the experience of the examining physician [5]. PSA, while specific to the prostate, is not cancer-specific and can be elevated in conditions other than PCa, such as benign prostatic hyperplasia (BPH) or prostatitis [6]. Consequently, the only definitive way to diagnose PCa is through histopathological examination of tissue obtained via biopsy. Several studies have highlighted the effectiveness of PSA screening in early PCa detection [7].

Once PCa is suspected, patients are typically referred for a transrectal ultrasound-guided biopsy (TRUS-Bx), the most common outpatient procedure for obtaining prostate tissue samples. During TRUS-Bx, 8 to 12 tissue cores are taken from areas of the prostate suspected to harbor malignancy. However, this method has notable limitations, including its tendency to underestimate the cancer grade and overdiagnose low-grade, clinically insignificant cancers, which may lead to unnecessary treatment [8]. Moreover, up to 50% of patients with a negative 12-core systematic biopsy may be diagnosed with PCa during follow-up, necessitating repeated biopsies if suspicion remains high [9].

In contrast, MRI-targeted biopsy has emerged as a more accurate diagnostic approach [10]. It involves performing multiparametric MRI (mpMRI) of the prostate prior to biopsy, allowing for the identification, localization, and detailed characterization of clinically significant cancer lesions. Combined biopsy (ComBx), which integrates MRI-targeted biopsies (TBxs) with additional systematic (mapping) biopsies (SBxs), is commonly used for PCa diagnosis. This approach utilizes real-time transrectal ultrasound to enhance prostate visualization and lesion localization based on the Prostate Imaging–Reporting and Data System (PI-RADS v2.1) [11].

This software-based fusion biopsy method is an increasingly utilized modality for MRI-targeted prostate biopsy, combining mpMRI images with real-time ultrasound to improve the accuracy of sampling lesions visible exclusively on mpMRI [12].

Although the effectiveness of MRI-targeted biopsy over TRUS-Bx has been well established in high-volume academic centers, data from lower-volume, non-academic institutions are scarce. This study, conducted in such a setting, evaluates the feasibility and diagnostic value of adopting ComBx as a primary tool for PCa detection in routine clinical practice.

## 2. Materials and Methods

This single-center, retrospective study analyzed a cohort of 500 consecutive men, aged 46 to 79 years (mean age 65), who underwent prostate biopsy at our Urology Department between 2017 and 2022. Patients were divided into two groups based on the biopsy technique used.

The first group consisted of 250 men who underwent TRUS-Bx, which involved systematic sampling of 12 cores from predefined regions of the prostate. TRUS-Bx was performed based on clinical indications, including elevated PSA levels or abnormal digital rectal examination (DRE) findings.

The second group comprised 250 men who underwent ComBx using the Koelis Trinity v. 4.8.2 (R1) software-based fusion system. This approach integrated MRI-targeted biopsy (TBx) with additional systematic sampling (SBx) to improve detection accuracy. In the ComBx group, MRI-targeted biopsies were obtained from suspicious lesions identified on mpMRI and classified as having a PI-RADS score ≥ 3. For each identified lesion, two to four targeted biopsy cores were obtained. Following the targeted biopsy, an additional eight to twelve systematic cores were collected to enhance sampling coverage. The decision to qualify a patient for biopsy in the ComBx group was based on mpMRI findings and adhered to the recommendations outlined in PI-RADS v2.1 and the European Association of Urology Guidelines. Patients without MRI-visible lesions or with lesions classified as PI-RADS 1 or 2 were not considered for biopsy.

Biopsy procedures were performed using a biopsy gun to obtain tissue samples. Patients with an initial PSA level above 50 ng/mL or those diagnosed with disseminated disease at the time of evaluation were excluded from the analysis.

The clinical data collected for each patient included the PSA concentration, prostate gland volume, and DRE findings. Histopathological outcomes from prostate biopsies were classified according to the International Society of Urological Pathology (ISUP) grading system, which assesses malignancy severity. For this study, clinically significant prostate cancer (csPCa) was classified as ISUP 2 or higher (Gleason Score ≥ 3 + 4 = 7).

The data are presented as numerical values, mean values with standard deviation (SD), and percentages. To evaluate the statistical significance of differences between the study groups, the Student’s *t*-test and Mann–Whitney U test were employed. For comparisons involving categorical data, Pearson’s chi-square test for independent groups was utilized, with additional post-hoc multiple comparison tests conducted if significant differences were observed. A *p*-value less than 0.05 was considered to be statistically significant. The statistical software of choice for all analyses was GraphPad Prism version 9.

Approval from the Medical University of Białystok’s bioethics committee was obtained in accordance with resolution number APK.002.439.2022 on 15 December 2022.

## 3. Results

In the TRUS-Bx group, the age of the patients ranged from 49 to 79 years, with a mean age of 65.1 years. PSA levels varied from 1.8 to 45.2 ng/mL, with a mean of 8.2 ng/mL. Prostate volumes ranged from 32 to 160 cm^3^, with a mean of 57.6 cm^3^. Palpable prostate abnormalities were detected in 22 cases. In the ComBx group, the age of the patients ranged from 46 to 76 years, with a mean age of 64.9 years. PSA levels ranged from 0.8 to 32.4 ng/mL, with a mean of 7.5 ng/mL. Prostate volumes ranged from 27 to 154 cm^3^, with a mean of 60.5 cm^3^. Palpable abnormalities were observed in 16 cases. There was no statistically significant difference between the TRUS-Bx and ComBx groups in terms of age, PSA levels before biopsy, prostate volume, or findings from DRE.

**Figure 1:**
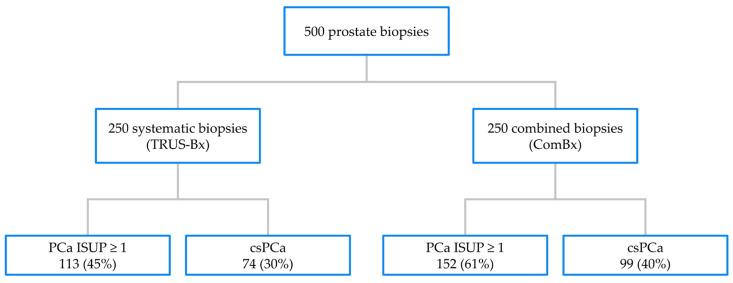
The detection rate of prostate cancer (PCa) in the studied population.

The efficacy of tumor detection between the ComBx and TRUS-Bx groups was compared, focusing on overall PCa and csPCa detection. In the ComBx group, PCa was confirmed in 152 cases (61%), while in the TRUS-Bx group, malignant tumor tissue was confirmed in 113 cases (45%). The difference was statistically significant (*p* < 0.01), as shown in Figure 1 and Table 1. Additionally, in the ComBx group, csPCa was confirmed in 99 cases (40%), compared to 74 cases (30%) in the TRUS-Bx group (*p* = 0.019).

**Figure 2:**
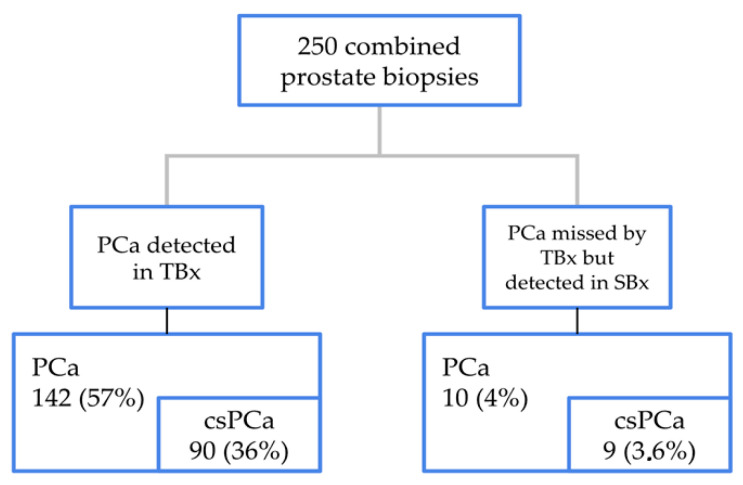
The distribution of TBx and SBx samples obtained during the combined biopsy, confirming the presence of malignant tumor tissue, with distinctions made between overall PCa and csPCa.

Histopathological results from targeted samples in the ComBx group revealed the presence of PCa in 142 out of 250 cases (57%). Among these, 52 cases (21%) demonstrated PCa ISUP 1, while 90 cases (36%) identified csPCa. In contrast, mapping samples identified malignancy in 108 out of 250 cases (43%). Among these, 10 cases were found in which PCa was not detected in the targeted biopsy, 9 of which were clinically significant, as shown in Figure 2. A statistically significant difference was observed in the frequency of overall PCa detection between the targeted and SBx samples during ComBx (57% vs. 43%, *p* = 0.002). Similarly, a statistically significant difference was found in the frequency of csPCa detection between the targeted and mapping biopsies (36% vs. 23%, *p* = 0.001). However, no statistically significant difference was noted in the frequency of clinically insignificant PCa detection (ISUP 1) between the targeted and systematic samples in the ComBx group (21% vs. 20%, *p* = 0.912), as shown in Table 2.

**Figure 3:**
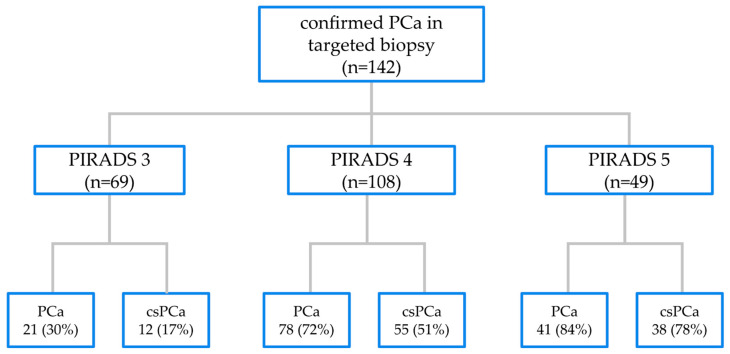
The distribution of targeted biopsy samples confirming the presence of malignant neoplasms, contingent upon the assessment of the PI-RADS classification for a given lesion observed in MRI examination, with distinctions made between overall PCa and csPCa.

In the ComBx group, 142 patients with confirmed PCa in targeted biopsy cores were analyzed based on the distribution of lesions according to the PI-RADS assessment. A total of 226 lesions were described in the mpMRI examination within this cohort. Among these, 69 (30%) were categorized as PI-RADS 3, 108 (48%) as PI-RADS 4, and 49 (22%) as PI-RADS 5. Histopathological results from cores obtained from 69 lesions assessed as PI-RADS 3 revealed tumor growth in 21 cases (30%). In 9 cases (13%), PCa with ISUP grade 1 malignancy was identified, while in 12 cases (17%), PCa with ISUP grade ≥ 2 malignancy was detected. In the 108 prostate areas assessed as PI-RADS 4, tumor growth was observed in 78 cases (72%). Among these, 23 cases (21%) exhibited PCa with ISUP grade 1 malignancy, and 55 cases (51%) exhibited PCa with ISUP grade ≥ 2 malignancy. In the 49 prostate areas assessed as PI-RADS 5, tumor growth was detected in 41 cases (84%). Of these, 3 cases (6%) showed PCa with ISUP grade 1 malignancy, while 38 cases (78%) exhibited PCa with ISUP grade ≥ 2 malignancy, as shown in Figure 3. The difference in PCa detection, both in ISUP grade 1 and ISUP ≥ 2 malignancies, in targeted cores based on PI-RADS assessment was statistically significant, as shown in Table 3.

## 4. Discussion

Accurately diagnosing PCa remains a challenge in modern urology. Traditional biopsy methods often lead to overdiagnosis of low-risk tumors and underdiagnosis of csPCa. The advent of mpMRI and fusion biopsy techniques has enabled a more targeted approach, enhancing diagnostic accuracy. In this study, we compared the efficacy of ComBx and TRUS-Bx, with a particular focus on their ability to detect csPCa. Our results indicate that ComBx significantly outperforms TRUS-Bx in overall PCa detection (61% vs. 45%) and shows a statistically significant advantage in identifying csPCa (40% vs. 30%). These results are consistent with previous studies, such as Ahdoot et al., who reported a 62% detection rate for ComBx, while the detection rate for csPCa with an ISUP grade of 3 or higher was 28% [8], and Ahmed et al., who found a 71% detection rate using fusion biopsy, with csPCa identified in 40% of cases [9]. Xu et al. reported a detection rate of 62.8% for combined targeted and SBx, compared to 55.9% for targeted biopsy alone and 52.6% for SBx [13]. The advantage of ComBx over TRUS-Bx was also emphasized by Fang et al. and Dorfinger et al. [13,14]. Integrating mpMRI into the diagnostic workflow enables targeted sampling of suspicious lesions. This reduces the risk of missing aggressive disease while minimizing the overdiagnosis of clinically insignificant tumors. Due to the limited diagnostic accuracy of systematic sampling in TRUS-Bx, mpMRI prior to biopsy has enabled targeted sampling of MRI-visible lesions. Combined biopsy refers to a prostate biopsy approach that integrates both systematic biopsy and targeted biopsy techniques to improve diagnostic accuracy [14,15].

MRI-targeted biopsy (TBx) has been widely recognized for its superior accuracy in detecting csPCa compared to systematic biopsy. Several studies have demonstrated that TBx alone enhances csPCa detection while reducing the identification of low-risk lesions. For example, Blas et al. reported a significantly higher detection rate of csPCa using TBx compared to TRUS-Bx (67.1% vs. 58.7%), while also reducing the detection of insignificant tumors (0.6% vs. 6.7%) [16]. This reduction in the identification of low-risk tumors is critical, as it decreases the likelihood of unnecessary treatments that may expose patients to adverse effects such as urinary incontinence and erectile dysfunction. Despite the advantages of TBx, relying solely on this approach may lead to missed diagnoses of csPCa, as demonstrated by the study conducted by Leow et al., in which systematic sampling provided an incremental detection rate of 10.4% for csPCa in biopsy-naïve patients and 2.4% in those with prior negative biopsies [17]. Oderda et al. assessed the additional diagnostic value of SBx following TBx, as well as the occurrence of PCa outside MRI-identified targets, and found that tumors outside MRI-targeted areas accounted for 33% of csPCa cases in the same lobe and 32% in the contralateral lobe, further reinforcing the necessity of systematic sampling [18]. Yao et al. reported that TBx alone had a significantly lower detection rate than ComBx in patients with PI-RADS 4 and 5 lesions (77.3% vs. 80.0%), with 71.4% (5/7) of the missed cases harboring csPCa [19]. Our findings align with these observations, as the omission of SBx in our study would have resulted in a clinically relevant number of missed PCa diagnoses—4% (10 cases), including 3.6% (9 cases) with undetected csPCa.

These findings highlight the indispensable role of taking additional samples alongside TBx in ensuring comprehensive cancer detection and minimizing the risk of underdiagnosis.

There is a consideration to reduce the number of SBx cores in favor of perilesional sampling. Recent studies have examined the utility of perilesional biopsies, which involve collecting additional tissue samples from the vicinity of MRI-detected lesions. Tomioka et al. found that incorporating perilesional biopsies into the TBx protocol significantly increased the detection rate of csPCa, particularly in patients with PI-RADS 4 and 5 lesions. The perilesional region was defined as the area within 10 mm of the MRI-identified lesion [20]. This indicates that an optimized biopsy approach incorporating perilesional sampling could further improve diagnostic accuracy and ensure more precise PCa grading while minimizing unnecessary tissue sampling.

Another consideration in biopsy strategy is the use of transperineal (TP) access as opposed to transrectal (TR) biopsy. The TR approach has been increasingly adopted due to its ability to access the anterior prostate region, an area challenging to reach with the TR approach. TP biopsy is associated with a lower risk of infectious complications compared to TR biopsies, reducing the incidence of sepsis and antibiotic-resistant infections, which further supports the advantages of ComBx [21]. However, no significant association was found between the biopsy approach and the overall detection rate of csPCa across all biopsy indications [22].

The use of mpMRI before biopsy has also prompted discussions regarding biopsy necessity in cases of negative imaging results. Studies by Pylväläinen et al. and Liang et al. suggest that in patients with nonsuspicious MRI findings, the decision to proceed with biopsy should be based on additional clinical factors such as age, PSA levels, and PSA density (PSAD). Specifically, a younger age, low PSA levels, PSAD < 0.15 ng/mL/cm^3^, and normal findings on DRE suggest that a prostate biopsy may not be required. A PSAD threshold of > 0.15 ng/mL/cm^3^ has been proposed as a key determinant for biopsy necessity in patients with negative MRI findings [23,24]. This approach could help reduce unnecessary biopsies while ensuring that high-risk patients, even with no apparent lesions on mpMRI imaging, receive appropriate diagnostic evaluation.

Despite its advantages, MRI-guided fusion biopsy is not without limitations. One major concern is the variability in MRI interpretation among radiologists, which can lead to inconsistent identification of target lesions and impact biopsy outcomes. Additionally, false-negative results may occur due to MRI’s limited sensitivity for certain cancer subtypes or small, non-visible lesions. Conversely, false-positive findings can lead to unnecessary biopsies, increasing patient anxiety and healthcare costs. The technical complexity of MRI-guided fusion biopsy, including operator dependency and the need for specialized equipment, further adds to the variability in diagnostic accuracy and outcomes [25]. 

The introduction of the PI-RADS system has significantly facilitated interpretation of prostate MRI. However, interobserver variability remains an issue. Looking ahead, the implementation of AI-based software holds promise for improving both detection and the assignment of PI-RADS categories, with some AI systems to date achieving better results than less experienced radiologists [26,27].

The superior detection rates of csPCa in the ComBx group highlight the importance of integrating mpMRI into standard biopsy protocols. This approach not only improves diagnostic accuracy but also has significant implications for PCa management, particularly in active surveillance and treatment decision-making. Additionally, MRI-guided biopsies provide a less invasive method for monitoring disease progression, minimizing the need for repeat biopsies and associated patient discomfort. ComBx also enables more precise identification of cases requiring treatment escalation, ensuring timely intervention for high-risk patients while avoiding overtreatment in those with indolent disease. Given these advantages, our findings support the growing consensus that fusion biopsy should be considered the gold standard for PCa diagnosis. A limitation of our study is its retrospective design and the relatively small sample size from a single center, which may affect its broader applicability and introduce potential bias in the results. Future research should focus on refining biopsy strategies, optimizing imaging techniques, and evaluating the cost-effectiveness of widespread mpMRI implementation. Moreover, the integration of artificial intelligence and machine learning in imaging interpretation could further enhance diagnostic precision, improving standardization and reducing reliance on operator expertise.

## 5. Conclusions

The combined fusion biopsy has demonstrated superior efficacy in detecting PCa, particularly in identifying a higher number of csPCa, compared to systematic biopsy. The findings from our study underscore the importance of incorporating multiparametric MRI into standard biopsy protocols, while future research should aim to validate these outcomes, explore the cost-effectiveness of MRI-guided biopsy, and reduce variability in radiologist interpretation to enhance diagnostic reliability.

## Figures and Tables

**Table 1:** Prostate cancer detection depending on the biopsy method employed.

	ComBx (n = 250)	TRUS-Bx (n = 250)	*p*-Value
PCa	152 (61%)	113 (45%)	*p* < 0.01
csPCa	99 (40%)	74 (30%)	*p* = 0.019

**Table 2:** The distribution of targeted and systematic (mapping) biopsy samples.

	Combined Fusion Biopsy		
	Targeted Samples (TBx)	Mapping Samples (SBx)	*p*-Value
PCa overall	142 (57%)	108 (43%)	*p* = 0.002
PCa ISUP 1	52 (21%)	51 (20%)	*p* > 0.05
csPCa	90 (36%)	57 (23%)	*p* = 0.001

**Table 3:** The distribution of targeted biopsy samples.

	PI-RADS 3 (n = 69)	PI-RADS 4 (n = 108)	PI-RADS 5 (n = 49)
ISUP ≥ 1	21 (30%)	78 (72%)	41 (84%)
ISUP ≥ 2	12 (17%)	55 (51%)	38 (78%)

## Data Availability

The dataset is available on request from the authors.
